# Glial Cell Expression of PD-L1

**DOI:** 10.3390/ijms20071677

**Published:** 2019-04-04

**Authors:** Priyanka Chauhan, James R. Lokensgard

**Affiliations:** Neurovirology Laboratory, Department of Medicine, University of Minnesota, Minneapolis, MN 55455, USA; guptap@umn.edu

**Keywords:** PD-L1, glia, central nervous system

## Abstract

The programmed death (PD)-1/PD-L1 pathway is a well-recognized negative immune checkpoint that results in functional inhibition of T-cells. Microglia, the brain-resident immune cells are vital for pathogen detection and initiation of neuroimmune responses. Moreover, microglial cells and astrocytes govern the activity of brain-infiltrating antiviral T-cells through upregulation of PD-L1 expression. While T-cell suppressive responses within brain are undoubtedly beneficial to the host, preventing cytotoxic damage to this vital organ, establishment of a prolonged anti-inflammatory milieu may simultaneously lead to deficiencies in viral clearance. An immune checkpoint blockade targeting the PD-1: PD-L1 (B7-H1; CD274) axis has revolutionized contemporary treatment for a variety of cancers. However, the therapeutic potential of PD1: PD-L1 blockade therapies targeting viral brain reservoirs remains to be determined. For these reasons, it is key to understand both the detrimental and protective functions of this signaling pathway within the brain. This review highlights how glial cells use PD-L1 expression to modulate T-cell effector function and limit detrimental bystander damage, while still retaining an effective defense of the brain.

## 1. Introduction

While most researchers now feel the term “immune privilege” is outdated and misleading when applied to the central nervous system (CNS), resident brain cells and their microenvironment do actively modulate immune responses [1]. Infection and insults to the CNS present unique challenges to host defense. Like other tissues, immune responses within the CNS are characterized by resident macrophage (i.e., microglial cell) activation, persistence of antibody producing B-cells, and retention of virus-specific memory CD8^+^ T-cells [2,3,4]. Infection-induced inflammation within the brain dramatically increases infiltration and migration of T-lymphocytes which play a critical role in antiviral defense. However, the chronic presence of interferon (IFN)-γ-producing lymphocytes may be damaging to this generally non-regenerating tissue [5]. Hence, in addition to the beneficial antiviral effects of turning on pro-inflammatory neuroimmune responses, suppression of inflammation is equally important in limiting tissue damage and preserving neurological function. Various immune checkpoints are specifically tailored to restrain exacerbated immune responses post-viral infection and a variety of inhibitory molecules contribute to T-cell regulation. One such checkpoint is the programmed death receptor-1 (PD-1): programmed death ligand-1 (PD-L1) pathway, belonging to the B7: CD28 family, that is vital in controlling interactions between cells involved in defense of the brain.

**Figure 1:**
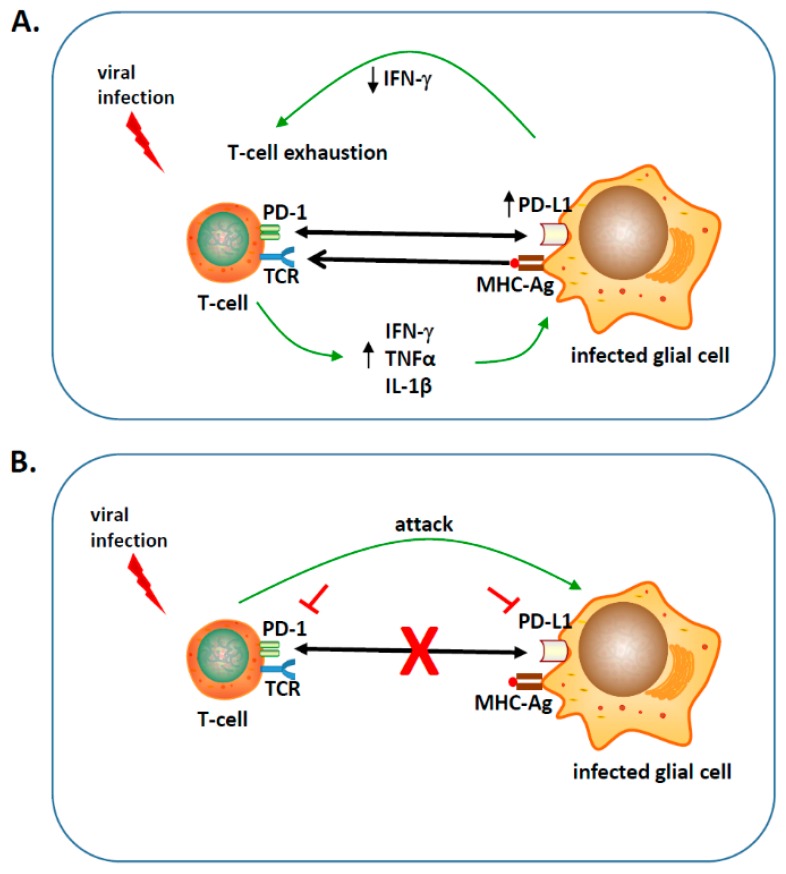
Glial cell modulation of T lymphocytes via the programmed death (PD)-1: PD-L1 pathway. (**A**) Engagement of the PD-1 receptor by its ligand PD-L1 results in functional suppression of brain-infiltrating T-cells. This results in controlled neuroinflammation, suppression of disease severity, and limited bystander tissue damage, but may also support persistent viral infection. (**B**) Blocking the PD-1: PD-L1 negative checkpoint results in the attack of infected glia by effector T-cells resulting in rapid viral clearance, but it may also promote reactive gliosis, exacerbated neuroinflammation, and disease severity. 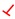 represents blocking either PD-1 or PD-L1 using inhibitors; 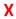 indicates blocking the PD-1: PD-L1 pathway; 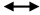 engagement of PD-1 to PD-L1; 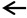 binding of major histocompatibility complex-antigen (MHC-Ag) to T-cell receptor (TCR).

PD-1: PD-L1 interaction plays a critical role in eliciting the inhibitory second signals that are required for functional suppression of T-cell responses, commonly referred as T-cell “exhaustion”. These exhausted phenotypes are characterized by decreased proliferative capacity, cytokine production, and cytotoxic effector functions (Figure 1) [6,7,8]. Correspondingly, the functional blockade of these interactions results in increased effector T-cell function [7]. 

Upregulation of the PD-1: PD-L1 pathway following infection and inflammation has drawn much attention over the past few decades, as these interactions limit immune-mediated tissue damage caused by over-reactive T-cells, especially at sites like the brain. It is also evident in models of CNS autoimmunity that PD-1: PD-L1 signaling dampens the auto-reactive T-cell responses protecting the CNS [9]. However, we need better understanding of the intricate balance between the antiviral neuroimmune responses necessary for efficient pathogen clearance, versus an acceptable level of bystander immune-pathological damage.

## 2. Expression of PD-L1 within the Brain

Microglial cells, astrocytes, oligodendrocytes, macrophages, dendritic cells, neurons, and a variety of other nonhematopoietic cell types including microvascular endothelial cells as well as epithelial cells, can express PD-L1 [10,11,12,13,14,15,16,17]. Cells in several other non-lymphoid tissues such as muscle, heart, placenta, renal tubular cells, ocular cells, and cancer lines also express PD-L1 [18,19,20,21]. In the CNS, microglial cells, originating from the yolk sac progenitors which seed the brain early during development, represent approximately 5–20% of the total glial cells in mice [22,23,24,25]. As CNS-resident immune cells, microglia are extremely sensitive to brain injury and disease, and function as a bridge between the CNS and immune system during autoimmune neuroinflammation [26]. Microglia serve as the critical cell type that not only initiates, but also regulates rapid immune responses against infections [27]. As a first line of host defense in the CNS, they modulate both innate (cytokine and chemokine production) and adaptive (T-cell activation) immune functions required for recruitment of infiltrating effector immune cells [28]. A basal level of PD-L1, is expressed on approximately 20% of microglia from uninfected mice, but induced expression is detectable on over 90% of the cells within one week following a viral brain infection [29]. During neuroinflammation both microglial cells, as well as astrocytes, upregulate PD-L1 along with MHC I and MHC II, suggesting a role for resident glial cells in limiting CNS pathology [30]. Given that microglia present antigen to T-cells (that express PD-1), expression of inhibitory co-stimulatory molecules like PD-L1 on microglia affect T-cell differentiation and function (i.e., production of pro-inflammatory cytokines). Besides microglial cells, astrocytes also play an important role in suppressing CNS pathology through PD-L1. It has been shown that human astrocytes up-regulate PD-L1 expression after treatment with pro-inflammatory cytokines which decreases activation of human CD8^+^ T-cells [31]. Astrocytes were also the first glial cells on which inducible major histocompatibility complex (MHC) II was reported [14]. Although, compared to microglia, expression on astrocytes is slower to develop and less robust, thus deeming them non-professional antigen presenting cells [32]. Importantly, it is clear that PD-L1 expression on glia can temper both reactive microgliosis as well as astrocytosis. Interestingly, B- and T-cells may themselves express PD-L1 that is further augmented upon activation with IFN-γ and IFN-β. Similarly, microglia also upregulate PD-1 upon infection induced inflammation [6]. Hence, we cannot exclude the possibility of microglia or T-cells interacting with themselves through the PD-1: PD-L1 pathway.

## 3. PD-L1 and Pro-Inflammatory Cytokines

PD-L1 up-regulation in the CNS occurs as a response to or in the presence of an inflammatory cytokine milieu (Figure 1). Upon viral infection, IFN-γ produced by brain-infiltrating lymphocytes results in the enhanced expression of PD-L1 on glial cells, whereas antigen experienced CD8^+^ T-cells express PD-1 [9,29]. Although cultured glial cells express low basal levels of PD-L1, its expression is upregulated upon IFN-γ, TNFα, or IL-1β- treatment, pro-inflammatory cytokines found during acute and chronic brain infection [2,31,33,34]. Undoubtedly, cytokine secretion by activated T-cells is responsible for long-term microglial activation and TNF-α production [2,34]. In the case of neuroinflammation, PD-L1 localizes within the areas of strongest inflammation and paradoxically acts as a “negative” regulatory feedback loop for keeping the anti-inflammatory milieu. The upregulation of PD-L1 limits CNS pathology through suppression of pro-inflammatory cytokines production from brain infiltrating T-cells. A preponderance of IFN-γ- vs. IL-17-producing T-cells in the CNS of PD-L1^−/−^ mice indicates this pathway mainly inhibits function of T_H_-1 cells rather than T_H_-17 cells [11]. Additionally, blocking the interaction of PD-1: PD-L1 between CD8^+^ T-cells and either primary murine microglia or astrocytes, resulted in increased IFN-γ and IL-2 production [29]. Similar observations of increased IFN-γ production by T-cells have been made in PD-L1^−/−^ mice [6], indicating a down-regulatory role of PD-L1. Increased expression of the IFN-γ inducible chemokine CXCL9 and its receptor following blockade of PD-L1 has also been reported, so this pathway may also negatively regulate chemokine expression [35]. Moreover, synergistic action between TNFα and IFN-γ in the infected brain increases nitric oxide (NO)-induced neurodegeneration. Inducible NO synthase (iNOS) is an effector molecule that synthesizes the free radical NO which is implicated in cytotoxicity and is closely associated with the pathological changes in various CNS disorders such as multiple sclerosis and its animal correlate, experimental autoimmune encephalitis (EAE) [36].

## 4. Role of PD-L1 in the Development of Brain-Resident Memory Cells (bT_RM_)

T_RM_s are a distinct class of memory CD8^+^ T-cells that are self-renewing, highly protective against subsequent infections, and permanently reside at sites of infection, disconnected from circulation [37]. Infiltrating T-cells, which persist long-term within an infected brain acquire a brain-resident T_RM_ (bT_RM_) phenotype and PD-L1 expression on activated glia contributes to this development. Notably, high PD-1 expression on bT_RM_ is an inherent property, ingrained and independent of both antigen and inflammation [38]. Analyses of post-mortem brain-derived T-cells also describes the expression of PD-1 on bT_RM_ [39]. However, the precise mechanisms and signaling pathways that drive its expression on bT_RM_ remain to be elucidated. The fact that T-cells in other organs, like the kidney, may not express PD-1 in spite of similar viral persistence as brain, indicates its tissue-restricted or organ-dependent induction dependent upon the microenvironment. Moreover, perennial PD-1 expression on bT_RM_ is regulated at an epigenetic level. During brain infection, the *PD-1* gene promoter first undergoes extensive demethylation to become accessible and then remains permanently demethylated [40]_._ Interestingly, despite PD-1 expression on bT_RM_, their effector functions were not impaired during infection, indicating they were not exhausted. Also, sustained expression of PD-1 on bT_RM_ even after the viral clearance indicates its role in controlling effector functions. Using both in vivo and in vitro studies, we have demonstrated a role for the PD-1: PD-L1 pathway in the development of bT_RM_. Using our murine cytomegalovirus (MCMV) infection model, we have shown that both PD-1 and PD-L1 knock-out animals display reduced number of CD8^+^ T-cells expressing bT_RM_ phenotype within chronically-infected brains, when compared to wild type animals [41]. Subsequent in vitro studies have also identified a role for PD-L1 on glia in promoting the development and retention of bT_RM_. Blocking PD-L1 on either microglia or astrocytes results in reduced expression of CD103, along with reduced CD127 on CD69^+^CD8^+^ T-cells (signature markers for bT_RM_) [42]. Another study using Theiler’s murine encephalomyelitis virus (TMEV) also demonstrated the critical role of PD-L1 in the maintenance of bT_RM_ post-acute encephalitis and viral clearance. The authors demonstrated that after resolution of acute encephalitis, effector T-cells developed into T_RM_ and persisted within brain in the absence of replicating virus. Further, T-cell adoptive transfer studies demonstrated that PD-L1 promoted maintenance of bT_RM_ in the CNS by restricting accumulation of pro-inflammatory PD-1^high^ CD103^-^ CD8^+^ T-cells. PD-L1 also plays an important role in controlling secondary virus challenge as lack of PD-L1 resulted in functional defect in bT_RM_-mediated virus control in knock-out animals [43]. Reports also indicate that treating bT_RM_ with anti-PD-L1 results in increased production of inflammatory cytokines [38,44]. Hence, PD-L1 not only influences the accumulation of virus-specific T_RM_ but also affects their cytokine production in the brain.

## 5. PD-L1: Mixed Blessings in CNS Disease

The CNS has unique features which make it a reservoir that allows for the persistence of a replication-competent virus, and brain cells harbor various persistent and latent viral genomes. These cells are generally long-lived with half-lives ranging from months to years and may be infected at a high frequency [45]. We know that overactive or chronic immune activation within the CNS can be detrimental to sensitive neurons resulting in focal immunopathology and corresponding neurological deficits. Several studies have examined the PD-1: PD-L1 pathway in CNS infections. In this review, we primarily discuss findings from various murine models. We have previously shown that PD-1 KO animals displayed significantly greater mechanical hypersensitivity than wild type mice during chronic murine retroviral (LP-BM5) infection, which was associated with significantly increased infiltration of T-lymphocytes, macrophages, and microglial activation [46]. In MCMV-induced brain infection there is chronic neuroinflammation, which persists within the post-encephalitic brain even in the absence of detectable viral antigen [2]. Using this model, Schachtele et al. demonstrated that activated brain-resident glial cells upregulate PD-L1 in response to IFN-γ produced by infiltrating T-lymphocytes and control antiviral responses through functional inhibition of effector CD8^+^ T-cells via the PD-1: PD-L1 pathway, thereby limiting the deleterious consequences of unrestrained neuroinflammation [29]. Extensive studies from other murine models illustrate the immunoregulatory role of microglial cells during chronic, persistent neuroinflammation [9,47,48,49]. PD-L1 expression on microglia, as well as on astrocytes and oligodendrocytes, has also been reported to negatively regulate T-cell activation and limit immune mediated tissue damage in models of multiple sclerosis (MS), as well as acute viral encephalitis [10,50]. In a human post-mortem study, expression of PD-L1 was observed on both microglia and astrocytes in the demyelinating disease MS brain lesions, but not in normal controls [31]. In a study by Hu et al., it was reported that when compared to naïve controls, microglia from EAE mice upregulated PD-L1 along with MHC II and CD86. Using an ex vivo co-culture, microglia from EAE mice inhibited antigen-specific CD4^+^ T-cell proliferation, as well as Th1 differentiation via NO (whose production was dependent on PD-L1) [48]. PD-L1 suppresses EAE severity and disease progression as demonstrated using PD-L1 knock-out animals, where PD-L1 deficiency leads to aggravated symptoms of EAE; as well as through adoptive transfer of M2-polarized microglia expressing PD-L1, which attenuated the severity of the established EAE, again demonstrating the important regulatory role of PD-L1 in neuroinflammation [51,52,53]. 

While anti-inflammatory responses within brain are undoubtedly beneficial to the host, establishment of a prolonged anti-inflammatory milieu may also lead to deficiencies in viral clearance. Using the gliatropic JHM strain of mouse hepatitis coronavirus, which results in acute encephalomyelitis in mice, Phares et al. have shown transient expression of PD-L1 by microglia and astrocytes, but higher, sustained expression by oligodendrocytes; suggesting a cell-type specific role for PD-L1 in CNS infection. Moreover, upregulated PD-L1 expression on microglia and oligodendroglia during active viral infection was found to suppress CD8^+^ T-cell function. The authors also highlighted the non-compensatory role of CD4^+^ T-cell help in optimizing CD8^+^ T-cell antiviral function at effector sites after PD-L1 blockade. The authors reported heightened neuroinflammatory responses via production of increased IFN-γ and granzyme B by CNS infiltrating CD8^+^ T-cells ultimately leading to more rapid clearance of virus from the CNS and decreased viral persistence in JHM-infected PD-L1^−/−^ mice [12,50,54]. However, this comes at a cost because PD-L1^−/−^ mice displayed a quicker onset of clinical symptoms (i.e., loss of axonal integrity) and a higher rate of morbidity and mortality (50% of the animals succumbed to infection) associated with sustained microglial cell and macrophage iNOS as well as TNFα expression. The absence of increased myelin loss suggested that PD-L1 does not protect oligodendrocytes directly from immune attack, but rather dampens ongoing immune activation. The authors further reported that modulation of T-cell effector function by PD-L1 leads to detrimental bystander damage to non-infected cells, despite more effective viral control [54]. This study highlights the role of PD-L1 in mediating protection from viral-induced immunopathology associated with encephalomyelitis. Similarly, Duncan et al. demonstrated upregulated levels of PD-L1 on microglia following IFN-γ and IFN-β stimulation during active TMEV-induced demyelinating disease (TMEV-IDD), an animal model of multiple sclerosis. Utilizing neutralizing antibodies, they demonstrated that PD-L1 suppresses TMEV-induced ex vivo production of IFN-γ, IL-17, IL-10, and IL-2 from CNS-infiltrating CD4^+^ and CD8^+^ T-cells, further supporting its regulatory function in T-cell control. However, an in vivo blockade of PD-L1 in SJL/J mice using a neutralizing antibody had minimal effects on viral clearance, but significantly exacerbated the severity of clinical disease symptoms (i.e., increased mortality) during the chronic autoimmune phase of TMEV-IDD, presumably through exacerbated immune-mediated damage [10]. Using a myelin oligodendrocyte model of EAE, it has been reported that adoptive transfer of wild-type encephalitogenic T-cells into PD-L1^−/−^ recipient mice results in enhanced severity of EAE disease in C57BL/6 animals. Moreover, PD-L1 deficiency converted the 129S4/SvJae strain from resistant to EAE-susceptible, again demonstrating a critical role of PD-L1 in limiting pathogenic effector T-cell responses [6]. Jun et al. reported a role for PD-L1 in the pathogenesis of herpetic stromal keratitis (HSK) caused by herpes simplex virus type 1 (HSV-1) infection of the cornea of mice. The administration of an anti-PD-L1 monoclonal antibody accelerated the development and severity of HSK, correlating with increased proliferation of HSV-1 specific CD4^+^ T-cells and IFN-γ production. In contrast, PD-L1 expression on 70% of the infected neurons (driven by IFN-β, not IFN-γ) ensuing rabies virus infection, prevents viral control resulting in neuroinvasion, enhanced CNS viral load, and mortality. Hence, in this case, PD-L1 deficiency rescues mice from fatal rabies virus infection [13]. In many neurotropic infections, PD-L1 expression on neurons provides a selective survival advantage to the virus. Thus, PD-L1 blockade therapy can be envisioned as a possible therapeutic approach in this context.

## 6. PD-L1, Glioma, and Checkpoint Blockade

Gliomas are major primary cancers in the CNS and there are complex networks involving both positive and negative immune regulation at the tumor site. Similar to pathogens exploiting the PD-1: PD-L1 pathway to establish persistence, expression of PD-L1 on brain tumors serves as an immune evasion strategy. Many human carcinomas and some T-cell tumors express PD-L1 on their cell surface [55]. Recently, PD-L1 expression has been shown to be upregulated on tumor-associated macrophages/microglia in the case of primary central nervous system lymphoma [56]. It has been reported that tumor cells expressing PD-L1 grow in wild-type mice but are regressed in PD-L1^−/−^ mice, data suggesting that PD-L1 on tumors may limit CD8^+^ T-cell clonal expansion and thereby attenuate tumor-specific responses [57]. These findings suggest that PD-1: PD-L1-mediated inhibitory signals grant tumors a selective growth advantage by inhibiting T-cell responses [6]. Immune checkpoint inhibitors function as tumor-regressing factors via the modulation of immune cell-tumor cell interactions by reactivating cytotoxic T-cells to work against cancer cells. Recently, Saha et al. have demonstrated in two glioma models that triple combination therapy of anti-cytotoxic T-lymphocyte- associated protein (CTLA)-4, anti-PD-1, and G47Δ-mIL12 (oncolytic HSV expressing murine IL-12) cured most of the mice with glioblastoma. This curative effect was associated with macrophage influx along with increased T-effector to T-regulatory cell ratios [58].

The first checkpoint inhibitor to be approved for the treatment of unresectable melanoma by the FDA was ipilimumab, which targets the CTLA-4 pathway [59,60]. Despite early achievements using CTLA-4 inhibition, the most widely used and successful immune checkpoint inhibitors are currently monoclonal antibodies which target PD-1 (pembrolizumab and nivolumab) and its ligand PD-L1 (Atezolizumab, Avelumab, and Durvalumab) [61,62,63]. These PD-1/PD-L1 inhibitory antibodies have been used as frontline treatment for various types of cancers, such as metastatic melanoma, non-small cell lung cancer (NSCLC), renal cell carcinoma (RCCs), and bladder or urothelial cancer [64,65]. Moreover, efforts are being made to consider them in numerous other cancer types, including breast, head and neck, and some advanced solid and hematological malignancies [58,66]. Over the past few years, immunotherapeutic approaches using checkpoint inhibitors have provided substantial improvements in the treatment of otherwise devastating malignancies for which conventional therapies have had very limited success [67,68]. Despite the huge success and efficacy of the anti-PD therapeutic response, it is limited to specific types of cancers, which is attributed to the insufficient and heterogeneous expression of PD-1 in the tumor milieu. Moreover, the response of individual patients and the susceptibility of particular cell types remain highly variable and difficult to predict [69]. Additionally, a targeted immune checkpoint blockade not only augments anti-tumor immunity, but also may induce specific, immune-related adverse events (irAEs) [70]. Off-target toxicities (e.g., neurologic toxicity) and innocent bystander inflammatory side effects are likely to account for much of the irAEs. Besides affecting the gastrointestinal tract, endocrine glands, skin, and liver, irAEs often involve the nervous system. Immune checkpoint blockade therapy can result in inflammation of the brain and can cause encephalitis and aseptic meningitis. Some patients receiving PD-1 pathway blockade therapy along with radiation against melanoma brain metastases reported seizure, cognitive changes, neurologic dysfunction, and perilesional edema as some of the toxicities [71]. The precise mechanisms behind irAEs are not clear, but appear to be mainly attributed to events mediated by antibodies, T-cells, and cytokine storms [63]. It is recommended that patients with high risk of irAEs be considered for treatment with immune checkpoint blockade only after weighing its risks and benefits.

## 7. Implications for PD-L1 Blockade Therapy in Clearing Viral Brain Reservoirs

Drugs targeting the PD-1: PD-L1 pathway, which have demonstrated their efficacy in stimulating T-cell responses, could potentially aid in the eradication of persisting virus [7]. Clinical trials are currently underway to determine whether intravenous administration of pembrolizumab is safe and tolerable for HIV-positive people. This is important because the potential exists for immune checkpoint inhibitor treatment-related toxicity and other adverse events, especially within the CNS. Striking a balance between immunity and inflammation within the brain is critical because lingering cognitive impairment seen in patients with HIV-associated neurocognitive disorders (HAND) on successful cART is believed to be related to persistent low-level CNS infection, accompanied by local immune activation [72,73]. Unfortunately, less is known about the safety of anti-PD therapy in patients with HIV infection, but a study by Davar et al., reported that this therapy could be safely given to patients with melanoma or NSCLC who also have HIV infection [74]. The preferential safe use of PD-1 or PD-L1 inhibitors over conventional drugs in randomized controlled trials including 4174 patients with advanced or metastatic cancers has been reported [75]. Hence, potentially toxic neuroinflammatory events associated with checkpoint blockade should be manageable in most cases [76,77]. Taken together, these findings suggest that blockade of the PD-1: PD-L1 pathway may provide the means to boost antiviral immunity and it appears to be safe in patients with chronic viral infections [63]. However, there is concern. It has been shown using an animal model that a PD-1 blockade antibody, when delivered systemically, was not able to enter the CNS during acute viral infection. This makes therapeutic targeting of PD-1: PD-L1 pathway within the CNS using antibody-based therapies challenging [43]. However, a recent review highlighted T-cell mediated antibody access thereby blocking the replication of neurotropic viruses [78]. Such novel insights provide key foundations for enabling the use of antibody therapy against various viral brain infections, as well as brain metastases.

## 8. Summary

Long-term persistent neuroinflammation is commonly seen during neurodegenerative diseases, stroke, and viral brain infection [79]. If not carefully managed, over-reactive, chronic immune activation can be detrimental to sensitive neuronal cells resulting in neurotoxicity, secondary neuronal injury, and subsequent neurocognitive dysfunction, as well as behavioral deficits [33]. Moreover, reactive microgliosis has been linked to synaptic dysfunction and loss, which affects learning and memory, suggesting extended neuroinflammation may be harmful to the brain [80]. This review focusses on understanding how elevated and sustained levels of a negative checkpoint molecule, PD-L1, regulates virus-specific T-cell responses and restrains chronic neuroinflammation. Expression of PD-L1 by microglia, astrocytes, oligodendroglia, and neurons provides accumulating evidence that it is a critical immune-modulator in CNS diseases. The distinct upregulation of PD-L1 by activated glia emphasizes the propensity of these CNS-resident cells to interact with lymphocytes. Therefore, elimination of the protective functions of PD-L1 to accelerate control of viral infection may be compromised by enhanced immune-mediated tissue damage. Still, modulation of this pathway holds promise in its therapeutic potential. A PD-1: PD-L1 checkpoint blockade may provide novel therapeutic approaches for enhancing antiviral activity and clearance of viral brain reservoirs.

## Abbreviations

PD	Programmed Death
CNS	Central nervous system
T_RM_	Tissue resident memory cells
CTLA	Cytotoxic T-lymphocyte- associated protein
NO	Nitric oxide
iNOS	Inducible NO synthase
EAE	Experimental autoimmune encephalitis
MCMV	Murine cytomegalovirus
TMEV-IDD	Theiler’s murine encephalomyelitis virus- induced demyelinating disease
MS	Multiple sclerosis
HSK	Herpetic stromal keratitis
HSV	Herpes simplex virus
NSCLC	Non-small cell lung cancer
RCC	Renal cell carcinoma
irAEs	Immune-related adverse events
HAND	HIV-associated neurocognitive disorders
IFN	Interferon
MHC	Major histocompatibility complex
Ag	Antigen
TCR	T-cell receptor
